# Testing Multivariate Adaptive Regression Splines (MARS) as a Method of Land Cover Classification of TERRA-ASTER Satellite Images

**DOI:** 10.3390/s91109011

**Published:** 2009-11-13

**Authors:** Elia Quirós, Ángel M. Felicísimo, Aurora Cuartero

**Affiliations:** Polytechnic School, University of Extremadura, 10071 Cáceres, Spain; E-Mails: equiros@unex.es (E.Q.); amfeli@unex.es (A.M.F.)

**Keywords:** multi-spectral classification, multivariate adaptive regression splines (MARS), area under the ROC curve (AUC), TERRA-ASTER image

## Abstract

This work proposes a new method to classify multi-spectral satellite images based on multivariate adaptive regression splines (MARS) and compares this classification system with the more common parallelepiped and maximum likelihood (ML) methods. We apply the classification methods to the land cover classification of a test zone located in southwestern Spain. The basis of the MARS method and its associated procedures are explained in detail, and the area under the ROC curve (AUC) is compared for the three methods. The results show that the MARS method provides better results than the parallelepiped method in all cases, and it provides better results than the maximum likelihood method in 13 cases out of 17. These results demonstrate that the MARS method can be used in isolation or in combination with other methods to improve the accuracy of soil cover classification. The improvement is statistically significant according to the Wilcoxon signed rank test.

## 1. Introduction

Conventional classification methods used in remote sensing have some basic problems due to the fact that they are not adapted to the real characteristics of image data. In addition, they lack proper configurations, and there is generally minimal user interaction.

Traditional remote sensing classification methods are divided into two large families. The first family is parametric, and includes the ML, bayesian methods, *etc.*; in this family, initial conditions (such as Gaussian distributions of reflectances or homoscedasticity) are not usually met in the remote sensing images. This means that the power of these tests is seriously undermined, and thus, classifications can be unnecessarily weak. The second family includes non-parametric methods (e.g., neural networks and classification trees). This second group addresses the problems posed by the first group by making no assumptions regarding reflectance distributions. Some of these methods have problems related to their “black box” format that seriously undermine any understanding of the relationship between initial variables and classification results, thereby greatly limiting the generalizalibility of the results.

According to [1], “parametric methods are those in which the decision rules are obtained from probability density functions for each class, whose parameters are estimated from samples collected for each learning category, without taking into account the others.” In this kind of classification, it is commonly assumed that the probability distribution functions for each class follow a normal multivariate distribution.

In addition to the assumption of a probability distribution that may be inappropriate for the data under analysis, parametric methods have added another substantial problem. Namely, if the data have a high dimensionality, many samples are required for the learning stage of these methods. Overall, the assumption of a normal spectral distribution is often violated, especially in complex landscapes. In addition, insufficient, non-representative, or multimode distributed training samples can further introduce uncertainty to the image classification procedure [2].

Many studies have shown that non-parametric methods provide better classification results. In studies such as [3], it is demonstrated that, even with small training samples, non-parametric classification algorithms provide better results than parametric ones. Also, [4] concluded that, under the assumption of overlapping training samples, a non-parametric algorithm is preferable as a classification method. With non-parametric classifiers, it is not required to assume that the data follow a normal distribution. No statistical parameters are needed to separate image classes. The decision function coefficients corresponding to separation boundaries between classes in representation space are obtained from samples of all classes, and such functions do not follow mathematically defined structures [1].

MARS is a non-parametric regression method in which no assumption is made regarding the functional relationship between dependent and independent variables. Instead, MARS builds this relationship from a set of coefficients and basic functions, which in turn are heavily influenced by the regression of the data. The operating method involves partitioning the area of entry into regions, each with its own regression equation [5].

This method was proposed by [6], and it is essentially an algorithm based on recursive partitioning and multi-stage regression that uses spline functions to align data with an arbitrary regression function [7]. Thus far, the MARS method has been widely used for predictive and simulation efforts, as in [8-11], and [12]. Other authors have compared this technique with other existing models. In this sense, [13] indicated that “MARS is better suited to model situations that include a high number of variables, non-linearity, multicollinearity and/or a high degree of interaction among predictors.” In addition, works such as [14] and [15] have praised the rigorous statistical basis upon which the MARS technique MARS has been built, and they have highlighted the clear advantage of MARS results as compared to the “black box” results generated by neural networks.

As shown in [16], the MARS method can be extended to handle classification problems. This study analysed two types of classifications. The first type was comprised of those that included only two classes; in this case, the MARS method without any variant is solid and competitive. A second type of classification with more than two classes was also considered; in this second case, the use of a hybrid method called PolyMARS was recommended. We do not detail the latter technique because in our case, classification is carried out by means of a pairwise procedure that corresponds to the first of these two types [16].

It should be emphasised that [17] and [18] included the MARS method in the neural multilayer networks classification group, but it appears that the MARS method has not yet been applied in the context of multi-spectral imaging classification efforts. In fact, [18] refers to the MARS and autonomous classification engine (ACE) methods by expounding that “we believe that these methods will have a place in classification practice, once some relatively minor technical problems have been solved. As yet, however, we cannot recommend them on the basis of our empirical trials.”

Only [19] has applied the MARS technique to remote sensing data, but since the author of that paper worked with radar images, the work reported in [20] is not comparable to that in our study. That is, radar data are active data, while the data used in this study are multi-spectral passive optical images, also known as advanced space-borne thermal emission and reflection radiometer (ASTER) images.

The ASTER sensor was developed in an attempt to use detailed geological data to understand phenomena such as volcanic activities that can significantly impact the global environment [20]. However, the current implementation of this technology is substantially different than expected, with the images produced by this sensor having been used for several purposes other than geologic ones. For example, various studies have used ASTER scenes to characterise urban areas [21], use information from five TIR bands for the discrimination of agricultural crops [22], estimate the biomass of boreal forests [23], validate ASTER scenes for the geometric reconstruction of cloud masses [25], investigate glacier geometries and movements [24,25], predict natural hazards [26,27], and detect soil temperatures [28,29]. Finally, it must be highlighted that [30] employed ASTER image to identify different types of land uses and also compared two types of classifiers.

On the other hand, on how to evaluate the accuracy of the classification, a receiver operating characteristic (ROC) curve is a 2D graph representing both the specificity and the sensitivity of a binary (*i*, *j*) classifier. The sensitivity of a classifier is the probability of classifying an item as belonging to class *i* when it actually belongs to class *i*. The specificity is the probability of classifying an item as not belonging to class *j* when it actually does not belong to class *j*.

AUC provides a measure of how well a classification algorithm performs. [13] pointed out that AUC summarises performance across all possible thresholds and is independent of balance among classes.

ROC curves are generated by varying a threshold across the output range of a scoring model and then observing the corresponding classification performances. This graph is necessary to obtain the AUC statistic. The AUC statistic has an important property; namely, the AUC of a classifier is equivalent to the probability that classifier will rank a randomly chosen positive instance higher than a randomly chosen negative instance [31]. A random guess classifier will produce a diagonal line on the ROC diagram with an area of 0.5. Hence, the AUC of a realistic classifier lies between 1 and 0.5. The high value of 1 corresponds with an ideal classifier, so the closer the AUC statistic is to 1, the better the classification is expected to be.

[32,33] concluded that the AUC statistic is more of a discriminating measure than an accuracy statistic. The use of the AUC statistic has been widely extended in predictive modelling techniques. However, only a few remote sensing works, such as [34] or [35], have used AUC for evaluating classification performance. This study uses the AUC statistic to evaluate three classification methods.

In brief, the aim of this study is to evaluate the MARS algorithm as a remote sensing classifier. For this purpose, the same TERRA-ASTER scene was classified by MARS and by two classical remote sensing classifiers (the ML and parallelepiped methods) to compare class probabilities derived from the AUC statistic. Section 2 describes the study's framework and data processing methods. Section 3 introduces the classification methods, and section 4 analyses and discusses the results.

## 2. Materials

### 2.1. TERRA-ASTER scene

**Figure 1.**
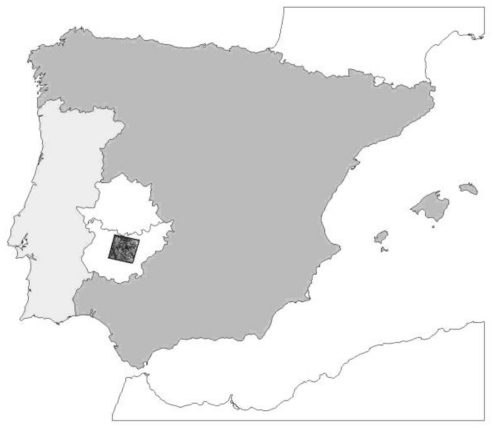
Location of the Extremadura test area in southeastern Spain.

The test area was a roughly 60 × 60 km area in the Spanish province of Badajoz, which is located in Extremadura in southeastern Spain (Figure 1). Elevation in this area ranges from 77 to 855 m, with an average of 360 m. The area was captured by the ASTER sensor on 4 August 2000.

The platform is composed of three different subsystems. First, the visible and near-infrared (VNIR) has three bands with a spatial resolution of 15 m and an additional backward telescope for stereoscopic use. Second, the shortwave infrared (SWIR) has six bands with a spatial resolution of 30 m. Finally, the thermal infrared (TIR) has five bands with a spatial resolution of 90 m. Each subsystem operates in a different spectral region with its own telescope [36]; see Table 1.

The ASTER data can be downloaded free of cost from the website http://asterweb.jpl.nasa.gov/.

### 2.2. Vegetation map

**Figure 2.**
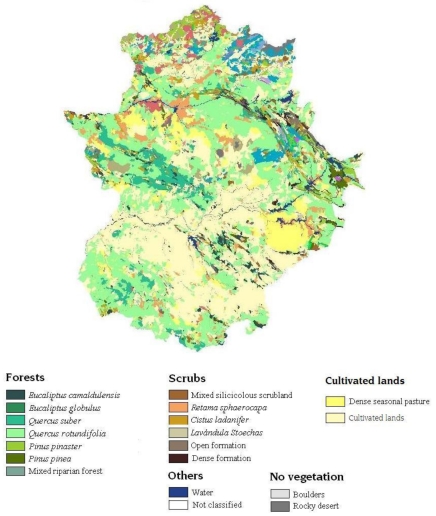
Extremadura forest map (EFM).

The Regions of Interest (ROIs) used in the classification process were obtained from the Spain forest map (see Figure 2). This map provides a polygonal distribution of territory based on homogeneous vegetation units characterised by information on vegetation type. This study used a version synthesised by the Extremadura Regional Government in 2001 [37]. This version includes the main forest categories groups existing in the region. Taking only the area under study into account, a total of 18 categories must be analysed (see Table 2).

### 2.3. Software

All operations were performed using a variety of softwares as ENVI (image processing), ArcView and ArcGIS (geographic information systems), SPSS (statistical analysis), and MARS (predictive modelling).

## 3. Methods

In this study, the data were pre-processed prior to any classification procedures (see Section 3.1). A brief introduction to the MARS algorithm is presented before developing different classifications (see Section 3.2.). Two different processes were performed in this study. On the one hand, we generated three traditional classification maps using the ML, parallelepiped and MARS algorithms (see Section 3.3.). On the other hand, class probability maps were also obtained using these three algorithms; their accuracies were calculated using the AUC statistic (see Section 3.4.).

### 3.1. Image preprocessing

Two different operations were implemented on the original image before starting classification processes. First, regardless of whether the data included L1-B images, that is, radiometrically calibrated and geometrically registered images, an additional geometric registration was performed using nine ground control points. These points were previously measured using GPS techniques.

**Figure 3.**
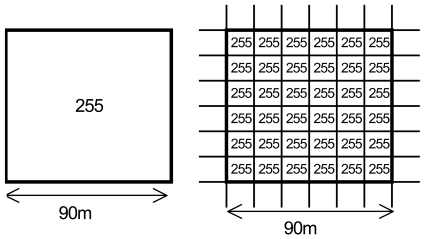
Re-sampling example for TIR bands.

Second, we re-sampled the SWIR and TIR bands to obtain the same geometric resolution for the entire image. With this action, no alteration was introduced to the original pixel information because the re-sampling was performed from a larger pixel resolution up to a smaller pixel resolution (see Figure 3). This re-sampling was necessary because the classifier requires homogeneous data to operate, and this method is simpler than introducing different band resolutions for different classifier calculations.

After these two processes, the image data were ready for classification.

### 3.2. Introduction to the MARS algorithm

The MARS model is a spline regression model that uses a specific class of base functions as predictors in place of the original data [38].

A spline is a special function defined piecewise by polynomials, and it is used to refer to a wide class of functions that are used in applications requiring data interpolation. [16] defined the cubic spline as a function that has first and second continuous derivatives. Cubic splines are also called basic functions.

On the other hand, basic functions have a key attribute known as a “knot”. A knot marks the end of one region of data and the beginning of another [38]. The number of knots and their placement are fixed for regression splines, and in the MARS procedure, knots are determined by a search that occurs both forwards and backwards in a stepwise fashion. First, MARS generates a model with an excessive number of knots; then, knots that contribute least to the overall fit are eliminated.

Basis functions are used to search for knots; these functions serve as a set of functions representing the relationship between the predictor variables (*X*) and the target variable (*y*):

(1) $$y=f(x)=\beta_{0}+\sum_{m=1}^{M}\beta_{m}h_{m}(X)$$

This function consists of an interceptor parameter *β_0_* and the weighted sum of other basic functions *h_m_(X)*.

MARS uses what [16] denotes as “reflected pairs”. These are linear basic functions of the form *(x − t)*_+_ and *(t − x)*_+_, with *t* being the knot.

The MARS procedure is divided into three steps. First, a forward algorithm selects all possible basic functions and their corresponding knots. Second, a backward algorithm eliminates all basic functions in order to generate the best combinations of existing knots, and finally, a smoothing operation is performed to obtain continuous partition borders.

1. The selection of basic functions from the initial set is achieved by determining a constant function *h_0_*(*X*) = *1* so that all functions from set C are candidates. New pairs of functions are considered at each stage until the model has the maximum number of terms set by the user at the beginning of the process.
2. The backward removal is performed by suppressing those model terms that contribute to a minimal residual error. This stage consists of reducing the complexity of the model complexity by increasing its generalisability [7]. This process can be conducted by means of generalised cross-validation (GCV).
  (2) GCV(λ)=∑i=1N(yi−^fλ(xi))2(1−M(λ)/N)2
  With this GCV function, the optimum number of model terms (*λ*) can be estimated with:
  (3) M(λ)=r+cK
  The value *M(λ)* is the effective number or parameters in the model, and it is expressed in terms of *r* (*i.e.*, the number of linearly independent basic functions) and *K* (*i.e.*, the number of knots selected in the forward process).
  The process stops when the number of model terms reaches *GCV(λ)*.
3. Finally, smoothing is necessary for removing discontinuities within regional borders and ensuring the continuity of first and second derivatives.

[16] included two possibilities for classification with MARS. The first one relates to pairwise classification in which the output can be coded as 0 or 1, thereby treating classification as a regression. The second possibility involves more than two classes, with the classification serving as a hybrid of MARS called PolyMARS as expounded in [39]. This study adopts the first technique.

### 3.3. Classification maps

In this process, 17 out of the total 18 categories were considered for classification. The “agricultural cultivations” class (cod 534) was excluded due to its heterogeneity. All the crops of dry regions and irrigated regions are incorporated into this EFM category so that very different spectral responses are included. Considering this class would cause serious errors in classification processes.

The original format of the EFM categories was the ArcInfo exchange coverage extension “*.e00”. Thus, an import operation became necessary to convert all classes into “shp” format. This process was performed using both ArcInfo and ArcView software.

The second preliminary operation, as referred to above, was to reduce all “shp” polygons through a buffer of −100 m in order to purify the ROIs. Not all polygons were chosen for the classification task. As shown in Table 3, the area of the ROIs fluctuate between 78.18% of the total area for class 221 and 31.89% of the total area for class 454.

Thus, we relied on a variety of ROI sizes to confirm whether there was relationship between training size and final classification results. As shown in Table 3, the smallest training size was for class 26, and the greatest size was for class 45.

This study uses the ML, parallelepiped, and MARS methods. A brief summary of the properties of each of these classifiers is given in the next section.

#### 3.3.1. ML classification

ML is the most popular parametric classification method used in remote sensing. This method assigns observation X to class ωI if the function gi(X) is larger than any other *g_j_(X)* for all *i≠j* so that:

(4) $${\text{g}}_{i}(X)=-\frac{1}{2}Ln(\left|\sum_{i}\right|)-\frac{1}{2}{\left(X-\mu_{i}\right)}^{T}\sum_{i}^{-1}\left(X-\mu_{i}\right)$$

#### 3.3.2. Parallelepiped classification

The parallelepiped classification method is a non-parametric method. It is one of the simplest classification methods based on a radiometric model rather than on the measurement of distances or probabilities [40]. The parallelepiped–like subspace is defined using training samples, and its boundaries can be determined in several ways, such as maximum or minimum pixel values, a corresponding class, a multiple of the standard deviation, and so on. The decision rule simply checks whether the point representing a pixel in the feature space lies inside any of the parallelepipeds [41]. The Boolean operator of the decision rule is based on the standard deviation.

#### 3.3.3. MARS classification

**Figure 4.**
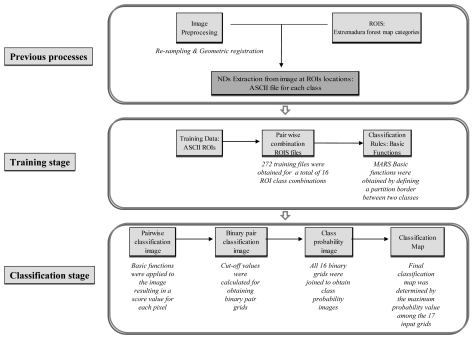
Work flow for the MARS process classification.

In order to monitor the whole battery process more effectively, a chart diagram is displayed, summarizing the methodology for MARS classifications (Figure 4).

**Figure 5.**
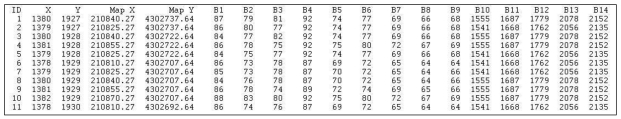
Fragment of the ROI ASCII export file.

For MARS classification, it is necessary to know the digital numbers (DNs) of pixel values at all 14 bands. Thus, once all shp ROI polygons were superimposed onto the ASTER scene, we performed an ASCII exportation using ENVI software. The 17 resulting files contained image and map coordinates of all pixels inside the corresponding ROI and their corresponding DN values for all bands. See Figure 5.

First, the image must be classified by discriminating each class from the rest. The class with which all other classes were compared was called the “fixed class”, and the fixed class was compared with the “comparing class”. Next, these classifications were merged into a unique probability image per class, and finally, the probabilities per class were joined into a final classification map. Based on this premise, the classification process was designed as follows.

##### a) Training stage

As discussed in [42], training is the classification stage used to estimate the parameters of the classifier algorithm; these parameters were the equations that defined partitions in multi-spectral space. The present study used the basic functions obtained by MARS for the multi-spectral space partition in each pair of classes.

###### a.1. Pairwise combination of ROI ASCII files

The pairwise combinations of ROI files were developed using SPSS. A new variable was introduced to distinguish between the fixed and comparing classes. This variable was assigned the value 1 for the fixed class and 0 for the comparing class. Overall, 272 training files were obtained, for a total of 16 ROI class combinations.

###### a.2. Obtaining basic functions

All 272 training files were introduced into the MARS software, and basic functions were obtained by defining a partition border between the two classes. Before validating these basic functions, they were applied to the input data again to verify that they could discriminate between class training data and that they could consistently extrapolate image holes for discriminating classes. This validation process was developed using the AUC statistic, and it was assumed to show the degree of adjustment of the MARS model relative to the input data.

##### b) Classification stage

Once the basic functions were obtained from the training data, the second stage consisted of applying them to the entire image in order to perform the actual classification.

###### b.1. Application of pairwise basic functions to the entire scene

Basic functions were introduced into an ArcInfo macro language (AML) file. This AML file was programmed to apply MARS basic functions to the image, resulting in a probability value for each pixel. This value indicated the probability of belonging to the fixed class (i.e., higher probability values) or to the comparing class (i.e., lower values).

###### b.2. Generating binary pair classification images

Until now, we have used 272 probability files for the combinations of all working classes. Now, we intend to generate a binary map in which the value 1 represents “pixel belongs to the fixed class” and value 0 represents “pixel belongs to the comparing class”, or equivalently, “pixel does not belong to the fixed class”.

The suitability scores obtained from MARS were not in the standard [0,1] interval. Thus, it was necessary to improve the application to calculate what some authors have called a “cut for best classification”. In our study, this value had to fulfil two conditions:

- Maximise correct classification probabilities
- Minimise incorrect classification probabilities

The application was programmed using SPSS software, and it counted false positive and false negative frequencies and subsequently changed the cut-off point in probability space. The process was similar to the one used for the ROC curve.

Once the cut-off value was calculated for all 272 probability files, the binary grids were generated using ArcInfo software.

###### b.3. Generating the class probability image

At this stage of the work, we had 16 binary grids per class. The purpose of the class probability image was to join all these binary grids.

All grids were added in ArcView, so the result was another grid with values ranging from 16 (if the pixel was always classified as the fixed class) to 0 (if the pixel had never been classified as belonging to the fixed class).

These values were transformed to the standard [0,1] interval dividing the grid by 16. Thus, the maximum probability value of 1 was assigned to those pixels that the MARS classifier always denoted with the same classification.

###### b.4. Obtaining the final classification image

The final operation was to join the 17 grids using ArcInfo software. This operation determined the class with the maximum probability value among the 17 input grids for each pixel.

### 3.4. Probabilities maps

At the same time that the classifications were calculated, a probability map was generated for each class. This allowed us to conduct an exhaustive evaluation of the method's accuracy in predicting classifiers.

#### 3.4.1. The ML probability maps

While performing classification processes, ENVI software allows for the calculation of rule images. Rule images for ML classifiers are grids containing the discriminant function expressed in [42] for multivariate normal class models:

(5) $$g_{i}(x)\ln p(\omega_{i})-\frac{1}{2}\ln\left|\sum_{i}\right|-\frac{1}{2}{\left(x-m_{i}\right)}^{t}\sum_{i}^{-1}\left(x-m_{i}\right)$$

The rule image obtained for this study contained values in the interval [−300,000, 100] with a heterogeneous distribution. This property impeded the use of rule images for the development of probability maps.

An alternative probability map was calculated by following the same pairwise method used for the MARS classification. We generated 272 binary ML classifications and combined them as explained in the MARS procedure to obtain the final probability map.

This option was also useful to validate the pairwise classification method because we did not stop at the class probabilities map but rather completed the entire process and thus obtained a final ML classification map; from now on, this will be called the pairwise ML classification map. If the pairwise classification process is valid, then the final pairwise ML classification map should be the same as the original ML classification map from Section 3.3.1.

#### 3.4.2. Parallelepiped probability maps

The ENVI rule images for parallelepiped classification provides for each pixel the number of bands that fulfils the parallelepiped condition. These pixel values were considered as probabilities with no transformations because the AUC statistic can be calculated with values not in the [0, 1] interval.

#### 3.4.3. MARS probability maps

Probability maps were calculated during the classification process, so it was not necessary to recalculate them.

## 4. Results and Discussion

### 4.1. Classification maps

**Figure 6.**
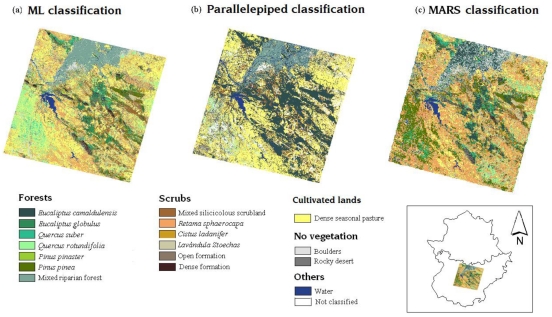
(a) ML classification map, (b) Parallelepiped classification map, (c) MARS classification map.

Figure 6 shows the three different classification maps obtained.

These classification maps cannot be used in and of themselves to evaluate accuracy. It is necessary to perform a separate accuracy study, and thus as mentioned before, ROC curves and AUC statistics were calculated for this purpose.

### 4.2. Comparison of classification maps

Accuracy assessments of the probability maps were implemented; Table 4 summarises the AUC statistic for each method.

Based on AUC statistics (Table 4), the MARS method obtains better results in 14 out of 17 classes, which confirms the suitability of this method for multi-spectral classification. We can conclude from the results shown in Table 4 that MARS is better than MV and PP but these are subjective interpretations. We performed three Wilcoxon paired-sample tests to estimate the P values for each comparison. Results are: MARS vs MV (P < 0.005), MARS vs PP (P < 0.001), MV *vs* PP (P < 0.001). We conclude that MARS is statistically a better method with a significance level better than 0.005.

## 5. Conclusions

This paper has presented the novel application of the MARS classification method to a conventional multi-spectral image. The results show that this method can be useful to improve some classifications. The main advantages of this method are that MARS is a non-parametric method that can be used without prior assumptions regarding the statistical distribution of the data. In addition, MARS is not severely affected by data collinearity, a common issue in satellite multiband imagery, and its functions are clear and transparent, especially when compared to the “black box” functions of other methods.

We compared the accuracy of various methods using the AUC statistic, an independent and objective test that can be applied to very different classification methods and that is independent of the cut-off classification threshold. The AUC statistic is a tool for evaluating classification performance that has been widely used in other disciplines, but only infrequently employed in remote sensing.

As the main conclusion of this study, MARS is a robust classification method that can be used in remote sensing without any disadvantages or apparent problems: its non-parametric nature, its transparency in terms of the relevant variables, and its adaptability to the data give it great potential as a multi-spectral classifier.

Our future work will focus on the application of hyperspectral data to MARS in order to deepen our analysis of its performance by using highly correlated bands and very large numbers of data.

## Figures and Tables

**Table 1.** Main characteristics of the ASTER sensor systems.

**Sub-system**	**Band n°**	**Spectral range (mm)**	**Spatial resolution (m)**	**Quantization levels**
**Visible & Near infrared (VNIR)**	1	0.52–0.60	15	8 bits
	2	0.63–0.69		
	3N	0.78–0.86		
	3B	0.78–0.86		

**Shortwave infrared (SWIR)**	4	1.60–1.70	30	8 bits
	5	2.145–2.185		
	6	2.185–2.225		
	7	2.235–2.285		
	8	2.295–2.365		
	9	2.360–2.430		

**Thermal infrared (TIR)**	10	8.125–8.475	90	12 bits
	11	8.475–8.825		
	12	8.925–9.275		
	13	10.25–10.95		
	14	10.95–11.65		

**Table 2.** Forest categories of area under study.

**Forest map cod.**	**Legend**	**Total area (Km^2^)**	**Percentage of the area under study**
**999**	Water	49.6	1.3%
**547**	Mixed silicicolous scrubland	47.5	1.2%
**534**	Agricultural land	2,422.9	61.4%
**507**	Mixed riparian forest	23.5	0.6%
**458**	Dense seasonal pasture	135.6	3.4%
**454**	Open formation	8.7	0.2%
**453**	Dense formation	8.6	0.2%
**337**	Boulders	2.1	0.1%
**329**	Rocky desert	42.2	1.1%
**309**	*Retama sphaerocapa*	109.2	2.8%
**303**	*Cistus ladanifer*	22.0	0.6%
**221**	*Lavandulas stoechas*	10.4	0.3%
**62**	*Eucaliptus camaldulensis*	194.7	4.9%
**61**	*Eucaliptus globulus*	10.1	0.3%
**46**	*Quercus suber*	53.5	1.4%
**45**	*Quercus rotundifolia*	786.0	19.9%
**26**	*Pinus pinaster*	1.4	0.0%
**23**	*Pinus pinea*	17.8	0.5%

**Table 3.** Regions of interest (ROI) used in the classifications.

**Forest map cod.**	**Legend**	**Category areas at image (km^2^)**	**ROI areas (km^2^)**	**ROI area percentages**
**999**	Water	49.6	33.3	67.19%
**547**	Mixed silicicolous scrubland	47.5	28.4	59.73%
**507**	Mixed riparian forest	23.5	11.6	49.18%
**458**	Dense seasonal pasture	135.6	52.2	38.47%
**454**	Open formation	8.7	2.8	31.89%
**453**	Dense formation	8.6	5.6	64.68%
**337**	Boulders	2.1	0.8	36.22%
**329**	Rocky desert	42.2	23.5	55.82%
**309**	*Retama sphaerocapa*	109.2	69.6	63.69%
**303**	*Cistus ladanifer*	22.0	10.6	48.33%
**221**	*Lavandula stoechas*	10.4	8.1	78.18%
**62**	*Eucaliptus camaldulensis*	194.7	140.6	72.25%
**61**	*Eucaliptus globulus*	10.1	5.7	55.93%
**46**	*Quercus suber*	53.5	26.0	48.48%
**45**	*Quercus rotundifolia*	786.0	544.2	69.23%
**26**	*Pinus pinaster*	1.4	0.7	52.24%
**23**	*Pinus pinea*	17.8	12.1	67.92%

**Table 4.** Area under the ROC curve (AUC) statistics.

**Forest map cod.**	**Legend**	**ROI areas (km^2^)**	**MARS**	**ML**	**Parallelepiped**

			AUC	AUC	AUC
999	Water	33.3	**0.952**	0.945	0.793
547	Mixed silicicolous scrubland	28.4	**0.852**	0.813	0.754
507	Mixed riparian forest	11.6	**0.936**	**0.936**	0.814
458	Dense seasonal pasture	52.2	**0.844**	0.714	0.687
454	Open formation	2.8	**0.978**	0.929	0.954
453	Dense formation	5.6	**0.985**	0.961	0.971
337	Boulders	0.8	0.963	**0.969**	0.791
329	Rocky desert	23.5	**0.890**	0.884	0.701
309	*Retama sphaerocapa*	69.6	**0.724**	0.699	0.670
303	*Cistus ladanifer*	10.6	**0.856**	0.826	0.728
221	*Lavandula stoechas*	8.1	**0.906**	0.898	0.657
62	*Eucaliptus camaldulensis*	140.6	**0.908**	0.856	0.834
61	*Eucaliptus globulus*	5.7	**0.949**	0.939	0.870
46	*Quercus suber*	26.0	**0.864**	0.841	0.766
45	*Quercus rotundifolia*	544.2	**0.688**	0.577	0.600
26	*Pinus pinaster*	0.7	0.957	**0.976**	0.903
23	*Pinus pinea*	12.1	0.952	**0.960**	0.924
